# Computational tibial bone remodeling over a population after total knee arthroplasty: A comparative study

**DOI:** 10.1002/jbm.b.34957

**Published:** 2021-10-18

**Authors:** Thomas Anijs, Sanne Eemers, Yukihide Minoda, David Wolfson, Nico Verdonschot, Dennis Janssen

**Affiliations:** ^1^ Orthopedic Research Laboratory Radboud University Medical Center, Radboud Institute for Health Sciences Nijmegen The Netherlands; ^2^ Department of Orthopedic Surgery Osaka City University Graduate School of Medicine Osaka Japan; ^3^ DePuy Synthes Joint Reconstruction, WW Research & Development Leeds UK; ^4^ Laboratory for Biomechanical Engineering University of Twente, Faculty of Engineering Technology Enschede The Netherlands

**Keywords:** bone remodeling, DEXA, finite element analysis, proximal tibia, total knee arthroplasty

## Abstract

Periprosthetic bone loss is an important factor in tibial implant failure mechanisms in total knee arthroplasty (TKA). The purpose of this study was to validate computational postoperative bone response using longitudinal clinical DEXA densities. Computational remodeling outcome over a population was obtained by incorporating the strain‐adaptive remodeling theory in finite element (FE) simulations of 26 different tibiae. Physiological loading conditions were applied, and bone mineral density (BMD) in three different regions of interest (ROIs) was considered over a postoperative time of 15 years. BMD outcome was compared directly to previously reported clinical BMD data of a comparable TKA cohort. Similar trends between computational and clinical bone remodeling over time were observed in the two proximal ROIs, with most rapid bone loss taking place in the initial months after TKA and BMD starting to level in the following years. The extent of absolute proximal BMD change was underestimated in the FE population compared with the clinical subject group, which might be the result of significantly higher initial clinical baseline BMD values. Large differences in remodeling response were found in the distal ROI, in which resorption was measured clinically, but a large BMD increase was predicted by the FE models. Multiple computational limitations, related to the FE mesh, loading conditions, and strain‐adaptive algorithm, likely contributed to the extensive local bone formation. Further research incorporating subject‐specific comparisons using follow‐up CT scans and more extensive physiological knee loading is recommended to optimize bone remodeling more distal to the tibial baseplate.

## 1 INTRODUCTION

Total knee arthroplasty (TKA) is one of the most successful surgical interventions, but despite reduced revision rates, the number of primary TKA failures is increasing as a result of the aging population and the acceptance of TKA in younger patients.^1^ Two common causes of long‐term implant failure are aseptic loosening and periprosthetic fracture,^1^, ^2^ which are linked to stress shielding‐related bone loss as observed in longitudinal DEXA studies,^3^, ^4^, ^5^ in line with Wolff's law. Clinical DEXA studies typically display a significant spread in bone density changes, attributed to various sources of variation in patient characteristics, such as differences in initial bone mineral density (BMD), preoperative knee alignment, and subject body mass index (BMI).

For instance, initial mediolateral (ML) bone density distributions may depend on native knee alignment, since clinical studies have demonstrated a higher medial baseline BMD in varus knees, while valgus knees typically have greater initial lateral bone density.^3^, ^6^, ^7^, ^8^ Subsequently, knee alignment may be changed during surgery, leading to changes in joint loads and load transfer to the periprosthetic bone. Hence, a mechanical TKA alignment may lead to relatively more medial bone loss in constitutional varus knees, and more laterally concentrated bone loss in native valgus knees. This trend has been observed in numerous clinical DEXA studies.^3^, ^4^, ^6^, ^8^, ^9^, ^10^ A higher initial BMD has also been associated with greater (relative) proximal bone loss regardless of knee alignment, by a computational and a clinical study,^6^, ^11^ respectively, causing mean proximal BMD to converge to a fixed density range after 2 years.

Furthermore, clinical studies have demonstrated a positive correlation between subject BW measures and proximal BMD levels several years after TKA.^8^, ^9^, ^12^, ^13^ Conversely, no pronounced effect of age and sex on tibial bone loss has been reported. Since the age range of a primary TKA cohort is typically limited, different TKA studies have been unable to demonstrate age‐related BMD decline in the preoperative tibia and subsequent remodeling.^9^, ^14^ However, age‐related bone loss is generally more pronounced in postmenopausal women,^15^ accounting for higher baseline BMD levels found in male TKA patients compared with female patients.^14^ No significant differences in postoperative density changes were found by sex in various studies.^8^, ^12^, ^14^, ^16^, ^17^ One study reported significantly less bone loss in lateral and distal regions in male patients,^18^ potentially due to corrective alignment change related to preoperative varus deformity, as constitutional varus is more common in men than in women.^19^

Investigating the effect of various sources of variation on periprosthetic bone changes in a clinical setting in more detail would require long‐term follow‐up studies with large patient cohorts. An alternative way to gain more understanding about the relative effects of these parameters is through computational modeling. Previous finite element (FE) models have assessed periprosthetic tibial bone loss using strain differences,^20^, ^21^ and by subsequent modeled bone loss through strain‐adaptive bone remodeling.^11^, ^22^ Current strain‐adaptive remodeling theories have been established and refined based on femoral bone changes following total hip replacement,^23^, ^24^ but to our knowledge have not been validated before against clinical outcome in the tibia. In the current study, computational bone remodeling outcome in a TKA cohort was compared against results of a longitudinal clinical DEXA study in a different patient group.^25^, ^26^, ^27^ The computational results were furthermore used to investigate the relative effects of patient characteristics on periprosthetic bone remodeling in more detail.

## 2 MATERIALS AND METHODS

FE models were created using a custom‐made workflow,^11^ based on a Japanese lower limb CT data set of 26 tibiae from 14 subjects who were scanned prior to TKA surgery. The model setup consisted of the following consecutive steps: (a) CT scan processing, (b) FE mesh generation, (c) material property assignment, and (d) application of boundary and loading conditions.

In the initial processing step, knee alignment angles were measured and tibiae were segmented from the available CT scans, taken in supine position. The hip–knee–ankle (HKA) and tibial varus–valgus (VV) angles of each knee were measured in the anteroposterior (AP) view using CT scan annotations in Slicer 3D^28^; the HKA angle was defined as the angle between the femoral and tibial mechanical axes, while the VV angle was the angular offset of the joint line perpendicular to the tibial mechanical axis. Positive knee angles were directed toward varus alignment. Based on the measured knee angles, each knee was allocated to be in varus, neutral or valgus alignment. Knees were considered to be in varus if the HKA angle was greater than or equal to 3°. Subdivision between neutral and valgus knees was made based on the measured tibial VV angle; knees were assigned to be in valgus in case of a valgus tibial joint line (VV < 0°). Subject characteristics including the measured knee angles and resulting alignment distributions are indicated in Table 1.

**TABLE 1:** Subject characteristics indicated by mean (range)

Number of subjects (male/female)	14 (4/10)
Number of knees (male/female)	26 (7/19)
Subject age (years)	69 (60–76)
Subject body weight^a^ (kg)	57 (48–70)
Subject body height^a^ (cm)	156 (140–165)
Subject body mass index^a^ (kg/m^2^)	24 (18–31)
Hip–knee–ankle angle (degrees)	1.9 (−5.7 5.4)
Tibial varus–valgus angle (degrees)	3.7 (−2.4 to 8.0)
Number of varus knees (male/female)	13 (5/8)
Number of neutral knees (male/female)	12 (2/10)
Number of valgus knees (male/female)	1 (0/1)
Median tibial tray size (sigma RP sizing)	2.5 (1.5–4)
Bone coverage (%)	87 (77–97)

^a^ Data from 12 of the 14 subjects.

The tibiae were automatically segmented based on boundary enhancement filtering and graph cut optimization^29^; the resulting segmentation was manually adjusted using Slicer 3D in case incorrect local bone edges were detected. Surface meshes were generated from the obtained binary voxel masks^30^ and smoothed using curvature flow.^31^ The bones were subsequently aligned according to the mechanical axis,^32^ with the largest inertial axis being defined as the longitudinal axis and neutral internal rotation referencing the medial third of the tibial tubercle.^33^

Tibial alignment, resection planning, and tray size determination were performed relative to the mechanical reference frame. For each tibia, a size‐matched cemented cruciate‐retaining, rotating platform (RP), cobalt–chromium‐alloy (CoCr) implant (Sigma RP, DePuy Synthes, Warsaw, Indiana) was placed following mechanical implant alignment. This alignment was achieved by placing the implant according to the mechanical axes, resulting in 0° of postoperative HKA and tibial VV angles, regardless of constitutional deformity and anatomical joint line orientations. The applied loading conditions accounted for 3° external rotation of the femoral component relative to the posterior condyles, which was adopted to compensate for flexion and extension gaps.^34^ A posterior tibial slope of 5° was adopted, based on the general surgical recommendation for cruciate‐retaining implants to match the patient's anatomy up to 5° of posterior slope, and an average anatomical slope of 10° and 6° in neutral and varus knees, respectively, in a Japanese population.^35^

The tibial resection level was defined 8 mm distally from the lowest point of the highest condyle, and the internal/external rotation of the RP tray was optimized to maximize the coverage of the resected bone surface. The correct implant size was set to be the largest tray size which could be placed on the resection surface with a maximum overhang below 2 mm, in line with a reported tibial coverage study.^36^ Bone coverage, defined as the relative resection plateau surface area covered by the base plate, was computed to numerically assess the achieved implant position and indicated that realistic implant positions were achieved, since values were in line with results of previous clinical and computational studies.^13^, ^36^, ^37^ Established implant sizes and bone coverage ratios are also indicated in Table 1.

A cement layer was generated based on a 0.75 mm offset over the entire bone contact surface of the tibial component, and used as outline for resection of the tibia by applying a Boolean operation (HyperMesh, Altair Engineering, Michigan). The tibiae were distally resected 150 mm beneath proximal resection level. The proximal tibia was fixed distally in order to reduce computational cost in the final FE simulations. All parts in the assembly were (re)meshed using first order tetrahedral elements with a size of 2 mm and were connected using fixed contact definition.

**FIGURE 1:**
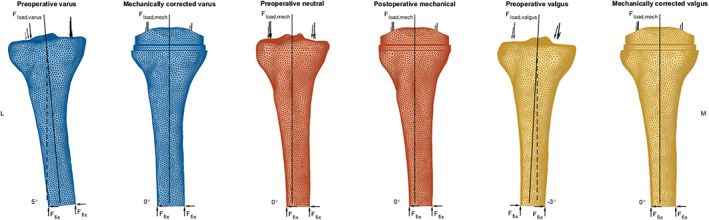
The anterior view of preoperative and postoperative FE model pairs of a native varus, neutral and valgus tibia, respectively, including applied loading and boundary conditions, and tibial mechanical axes and VV angles

Consistent preoperative and postoperative tibial bone meshes were used by reconstructing the intact preoperative models out of the two resected proximal bone parts, to allow for element‐wise evaluation of TKA‐related strain differences. The difference in alignment between preoperative and postoperative models, in case of native varus and valgus knees, was accommodated for by rotating the resected bone meshes toward their respective VV angles, consistent with the definitions used in the applied loading conditions, as depicted in Figure 1.

Bone material properties of the tibial meshes were assigned per element using corresponding CT intensities.^38^ The scan‐specific linear function between Hounsfield units (HU) and BMD was determined using a calibration method based on the intensities of air, fat and muscle tissue^39^; linear elastic properties of the calibrated bone densities were subsequently assigned using reported modulus–density relationships of cortical and cancellous bone, respectively.^40^, ^41^ Elastic moduli used for the CoCr tray, the polyethylene (PE) insert and the bone cement were 210 GPa, 588 MPa, and 2,551 MPa, respectively.

**FIGURE 2:**
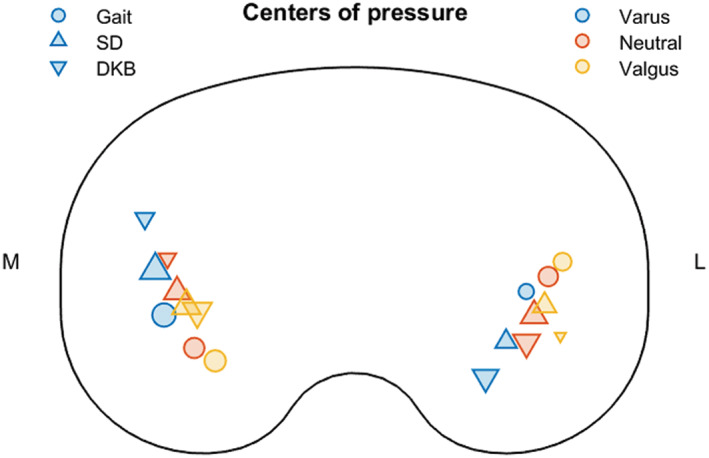
Schematic superior view of medial and lateral COP positions relative to the tibial tray during activity peak For Peer Review loads; markers are scaled based on the extent of the corresponding forces indicated in Table 2

Implant‐specific and alignment‐specific knee loading during physiological activity cycles was computed at the University of Denver using inverse dynamics,^42^ based on available in vivo loading and knee kinematics.^43^ Tibiofemoral (TF) peak activity forces of, consecutively, a gait, step down (SD), and deep knee bend (DKB) load cycle (Table 2) were applied at the centers of pressure (COPs) of the lateral and medial femoral condyles on the insert contact surface (Figure 2), to represent implant loading during daily activity. A tibial insert–tray combination in size 3 has been used to compute all implant load cases. Therefore, a size 3 insert was placed on the tray in each FE model, regardless of the used tray size. The used insert size is clinically compatible with tray sizes 2.5, 3, and 4, used in nine, eight, and five cases of the FE population, respectively, while the trays on the remaining four tibiae would be undersized in practice to match the insert sizes 1.5 and 2 following automated implant placement (Table 1). Forces were determined based on a subject body weight (BW) of 75 kg, and the extent of the applied forces was subsequently scaled to the available subject BW of the modeled tibiae. For the four tibiae of female subjects for which BW was not known, forces were scaled to the average female BW of the FE population (59 kg). Additionally, these four tibiae were simulated following the unscaled load cases of 75 kg to investigate the effect of load scaling on remodeling outcome.

**TABLE 2:** Numerical values of medial and lateral condyle forces during applied activity peak loads, given in percentage of subject body weight [%BW (N/kg·100%)]

Alignment	Activity	Medial condyle load (%BW)				Lateral condyle load (%BW)			
		*F*_med_	*F*_ant_	*F*_dist_	*F*_res_	*F*_med_	*F*_ant_	*F*_dist_	*F*_res_
Varus	Gait	−3	−15	232	233	−7	6	100	100
	SD	−9	9	294	294	−18	−3	145	147
	DKB	−9	6	114	115	−17	0	211	211
Neutral	Gait	14	−12	177	178	0	0	162	162
	SD	9	12	218	218	−9	−7	222	223
	DKB	3	6	95	95	−5	−7	231	232
Valgus	Gait	26	−8	194	196	9	−2	137	137
	SD	21	8	259	260	1	−4	175	175
	DKB	32	13	269	271	2	−6	36	37

In the preoperative models, the COPs of the forces, according to the subject's native deformity, were connected to the closest nodes on the proximal tibial surface using springs; the number of connected nodes was determined as function of the total related contact area, and spring constants were individually assigned based on distance and a compressive modulus of 9 MPa representing the intermediate articular cartilage.^44^ Neutral preoperative alignment was considered to be consistent with the planned mechanical implant alignment. In native varus alignment, a 3° HKA angle and a 5° VV angle were adopted in the preoperative situation, based on the constitutional varus HKA alignment found over multiple cohorts,^19^, ^45^, ^46^ in combination with an additional 2° tibial varus offset in the anatomical joint line.^47^ Valgus knees were represented preoperatively using a neutral 0° HKA angle in combination with a −3° tibial VV angle.

The averaged strain energy density (SED) after application of the three activity peak loads was considered as measure for bone strains during daily living. Subsequent iterative bone density changes were simulated using strain adaptive remodeling, with the difference between local preoperative and postoperative SED per unit bone mass, $S_{\mathrm{ref}}$ and S, respectively, considered as stimulus for density change in time $\left[\mathrm{d\rho}/\mathrm{dt}\right]$.^23^ If the relative local difference was lower than 35%, the stimulus fell into a *lazy zone* and no net remodeling was assumed. Outside of this range, the rate of local bone apposition or resorption was dependent on its available free bone surface a, representing the porosity and specific surface and determined based on the corresponding bone density ρ.^48^ Bone associated with greater free surface density a was assumed to be more responsive to changes in SED, since remodeling activity takes place at these free surfaces.

(1) $$\left[\frac{\mathrm{d\rho}}{\mathrm{dt}}\right]=\left\{\begin{array}{c} 0\\ a\left(\rho \right)\left\{S-1.35\cdot S_{\mathrm{ref}}\right\}\\ a\left(\rho \right)\left\{S-0.65\cdot S_{\mathrm{ref}}\right\} \end{array}\right.\begin{array}{c} \text{if}\;|S/S_{\mathrm{ref}}-1|<0.35\\ \text{if}\;S/S_{\mathrm{ref}}-1\geq 0.35\\ \text{if}\;S/S_{\mathrm{ref}}-1\leq -0.35 \end{array}$$

Definition of the local bone remodeling rate $\left[\mathrm{d\rho}/\mathrm{dt}\right]$ following the strain adaptive theory,^10^ as incorporated in the iterative postoperative FE simulations.

Simulations were time‐scaled using computer time units (CTU) defined relative to the maximum stimulus per iteration; each postoperative year was considered to correspond to 30 CTU. Used lazy zone and time conversion values were established in a study by Tarala et al.,^24^ in which simulated periprosthetic bone adaptations around a femoral hip implant were fitted to clinical data of a 2‐year clinical follow‐up study.^49^ Simulations incorporating the custom remodeling algorithms were conducted in MSC.MARC (MSC Software Corporation, Santa Ana, California).

**FIGURE 3:**
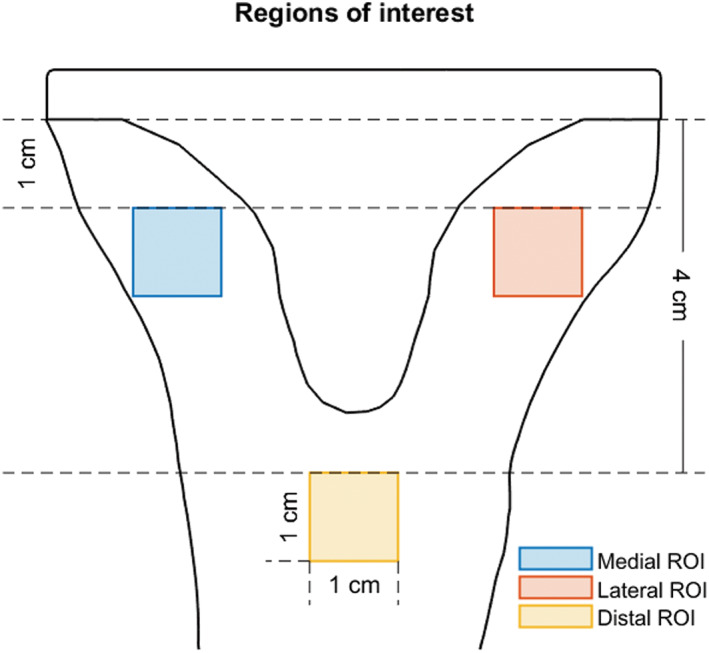
Schematic AP view of the ROIs, following the definitions of Minoda et al.,^25^ including reference distance measures. Medial and lateral ROIs were placed 1 cm distal to the baseplate within the medial and lateral cortex, respectively; the distal ROI was positioned 4 cm distal to the tray, medially centered following the ML position of the keel. Each ROI was 1 cm^2^

Two‐dimensional AP projections were made by mapping the element numbers of the FE models using a cubic voxel size of 0.2 mm^3^ in a three‐dimensional matrix,^30^ and subsequently taking the sum of the associated bone mineral content (BMC) values in AP direction. This method allowed us to efficiently compute virtual AP DEXA projections over a large number of time points, to allow for density comparison with existing longitudinal DEXA studies.^25^, ^26^, ^27^ The reported clinical densitometry data was measured in a different Japanese TKA cohort receiving the same cemented RP implant (Sigma RP), with follow‐up at 6, 12, 18, and 24 months;^25^ 3, 4, and 5 years;^26^ and final follow‐up at a mean of 11.4 years,^27^ respectively. The CT scans for the FE population and the densitometry data of the clinical population have been cleared for use in this study by two separate IRB approvals. BMC was determined in three tibial regions of interest (ROIs) of 1 cm^2^, defined relative to the position of the tray and according to the definition of Minoda et al.,^25^ illustrated in Figure 3, to directly compare numerical BMD outcome.

## 3 RESULTS

A comparison between available preoperative parameters in the different Japanese TKA populations used in computational and clinical remodeling is shown in Table 3. No significant differences in patient demographics were encountered, but preoperative medial ROI BMD was found to be significantly higher in the clinical DEXA scans, taken 2 weeks prior to TKA, than in the FE models.

**TABLE 3:** Comparison between preoperative parameters in the FE and clinical Japanese TKA populations

	FE population	Clinical population^27^
Follow‐up (years)	0–15	0, ½, 1, 1½, 2, 3, 4, 5, 11.6 ± 3.0
Number of knees	26	17
Sex (male/female)	7/19	5/12
Age (years)	68.7 ± 5.6	70.9 ± 6.8
Height (cm)	155.0 ± 7.0	152.4 ± 9.6
Body weight (kg)	55.9 ± 5.8	59.5 ± 9.3
Body mass index (kg/m^2^)	23.5 ± 3.6	25.6 ± 3.3
*Preoperative BMD (g/cm* ^*2*^ *)*		
Medial ROI	0.594 ± 0.162	0.854 ± 0.311^a^
Lateral ROI	0.466 ± 0.084	0.544 ± 0.147
Distal ROI	0.701 ± 0.160	0.656 ± 0.140

*Note*: Quantitative subject characteristics (mean ± *SD*) were assumed to be normally distributed in all subject groups, and were tested against difference using the two‐tailed *Z*‐test.

^a^ Statistically significant for *p* < .05.

**FIGURE 4:**
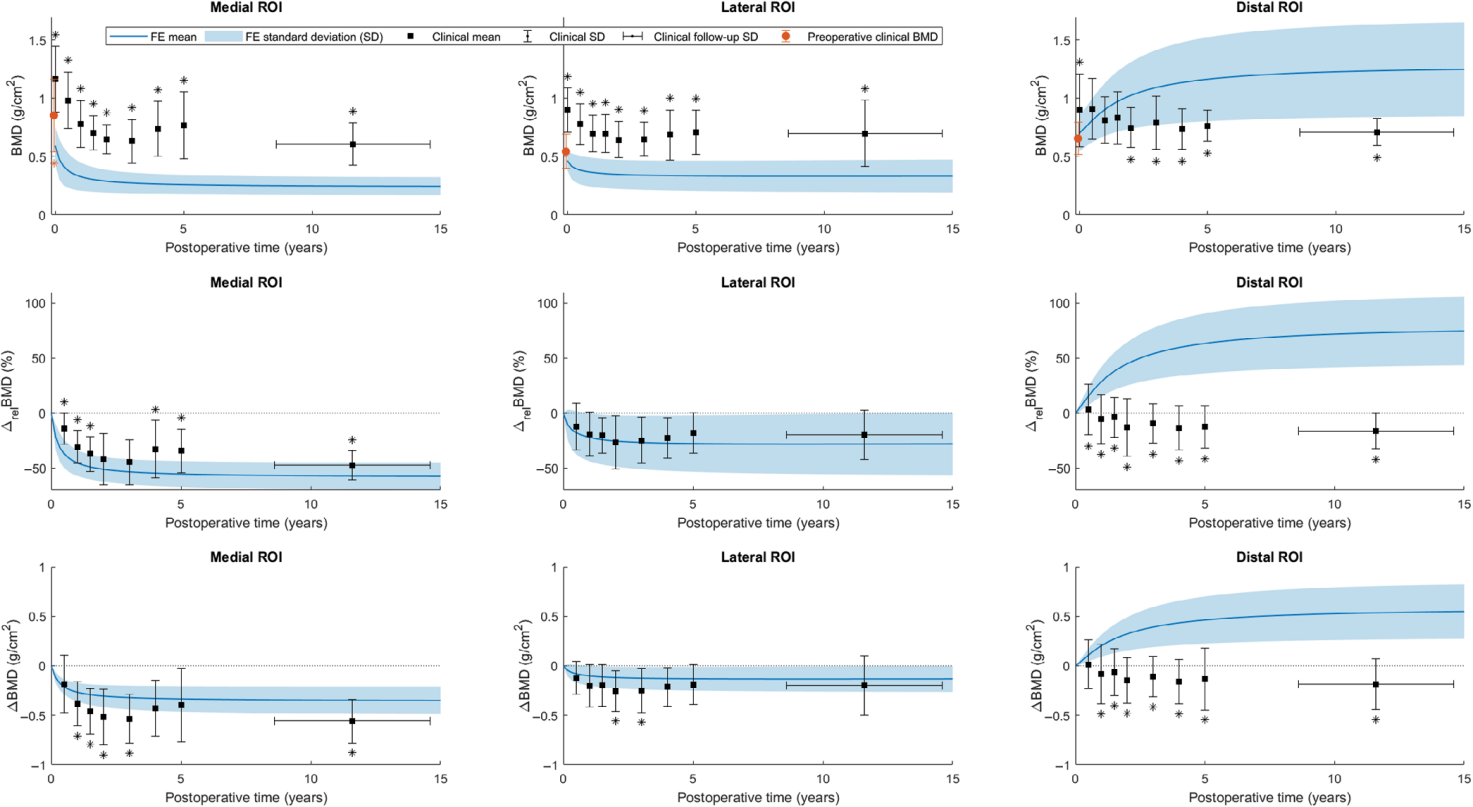
BMD outcome of computational (FE) and clinical remodeling (mean ± *SD*) over postoperative time in the three ROIs for different remodeling parameters. * = statistically significant difference between FE and clinical outcome for *p* < .05 following a two‐tailed *Z*‐test

Computational bone remodeling outcome over time was compared with reported clinical postoperative BMD outcome at different follow‐up time points in Figure 4 and Table 4. The most notable difference between clinical and computational results was the large increase in distal ROI BMD in the FE simulations, which was not observed clinically and was statistically significant in all considered remodeling parameters. Since all clinical baseline BMD values, taken 2 weeks after TKA, were considerably higher than corresponding clinical preoperative BMD and initial computational BMD, absolute densities remained significantly different between FE and clinical tibiae over postoperative time in the proximal ROIs. Despite significant differences in baseline BMD, the relative BMD decrease in the lateral ROI was not significantly different at any follow‐up time point; in absolute terms, clinical lateral bone loss was significantly greater at 2 and 3 years postoperatively. In the medial ROI, relative bone resorption was significantly smaller in the clinical population, while the absolute clinical bone loss was significantly greater at the majority of follow‐up time points.

**TABLE 4:** Regional BMD outcome (mean ± *SD*) of computational (FE) and clinical remodeling after TKA at different follow‐up time points

Follow‐up	Medial ROI		Lateral ROI		Distal ROI	
	FE	Clinical^27^	FE	Clinical^27^	FE	Clinical^27^
Baseline/2 weeks						
BMD (g/cm^2^)	0.594 ± 0.162	1.166 ± 0.282^a^	0.466 ± 0.084	0.899 ± 0.190^a^	0.701 ± 0.160	0.897 ± 0.314^a^
2 years						
BMD (g/cm^2^)	0.289 ± 0.099	0.649 ± 0.127^a^	0.347 ± 0.127	0.646 ± 0.156^a^	1.025 ± 0.290	0.749 ± 0.172^a^
Δ_rel_BMD (%)	−51.0 ± 11.4	−41.6 ± 16.0	−25.3 ± 23.7	−26.3 ± 17.6	44.9 ± 20.0	−13.0 ± 16.7^a^
ΔBMD (g/cm^2^)	−0.305 ± 0.113	−0.517 ± 0.281^a^	−0.118 ± 0.110	−0.253 ± 0.205^a^	0.323 ± 0.171	−0.148 ± 0.232^a^
5 years						
BMD (g/cm^2^)	0.264 ± 0.083	0.768 ± 0.287^a^	0.336 ± 0.131	0.709 ± 0.190^a^	1.156 ± 0.365	0.764 ± 0.132^a^
Δ_rel_BMD (%)	−54.5 ± 11.6	−34.2 ± 25.8^a^	−27.5 ± 26.0	−18.1 ± 21.0	62.3 ± 27.5	−12.3 ± 23.6^a^
ΔBMD (g/cm^2^)	−0.330 ± 0.131	−0.451 ± 0.372	−0.130 ± 0.121	−0.175 ± 0.203	0.454 ± 0.241	−0.181 ± 0.313^a^
11.6 (± 3.0) years						
BMD (g/cm^2^)	0.248 ± 0.080	0.608 ± 0.183^a^	0.333 ± 0.140	0.700 ± 0.284^a^	1.236 ± 0.404	0.711 ± 0.118^a^
Δ_rel_BMD (%)	−57.1 ± 12.4	−47.3 ± 13.3^a^	−28.1 ± 28.1	−19.6 ± 36.0	72.8 ± 31.1	−16.0 ± 16.6^a^
ΔBMD (g/cm^2^)	−0.346 ± 0.139	−0.558 ± 0.219^a^	−0.133 ± 0.131	−0.199 ± 0.301	0.534 ± 0.275	−0.187 ± 0.255^a^

*Note*: Difference between FE and clinical outcome was tested using the two‐tailed *Z*‐test.

^a^ Statistically significant for *p* < .05.

**FIGURE 5:**
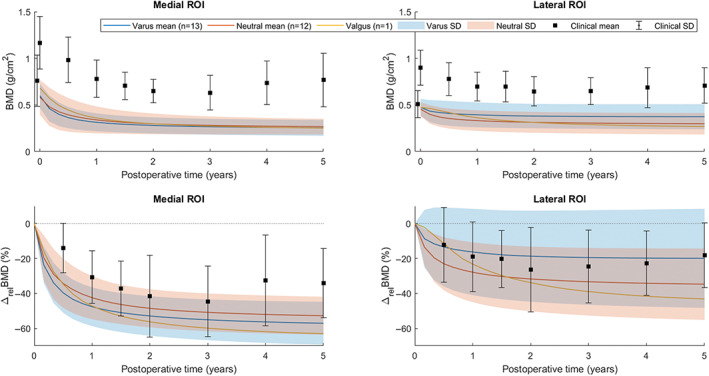
BMD outcome and relative BMD change of computational (FE) remodeling, differentiated by preoperative alignment, and clinical remodeling (mean ± *SD*) over postoperative time in the two proximal ROIs

The effect of alignment on periprosthetic bone changes was mainly observed in the lateral ROI, where a difference was seen between varus and neutral groups, with native neutral alignment cases displaying more bone loss at all time points (Figure 5). However, this difference did not become significant at any point in time when comparing relative and absolute remodeling outcome (*p* < .05). Despite slightly more initial medial bone loss in native varus knees, the mean BMD and associated variance of the two alignment groups became constant and almost equal again after the first 5 years. No considerable difference was found in the distal bone formation. The single native valgus case did not show a deviating trend from either of the other two alignment groups. No statistically significant difference was found between remodeling in male and female knees.

Other FE subject characteristics were tested against initial BMD, and against relative and absolute 15‐year ROI BMD difference (Table 5). Preoperative BMD values in all ROIs were positively correlated to each other, suggesting that relative density levels were equally distributed over the entire proximal tibia. Initial densities in all ROIs were significantly correlated to 15‐year density changes in the medial and distal ROIs, but not in the lateral ROI. The negative correlation coefficients over the medial ROI indicate that increased relative and net medial bone loss is encountered in preoperatively denser tibiae, while the positive coefficients found for bone changes in the distal ROI reveal an increase in distal bone apposition in higher density bones. Similar but less pronounced trends in medial and lateral bone remodeling were established for subject age at time of TKA, BW, and body mass index (BMI); most probably since these factors were positively correlated to baseline BMD, although not significant in most ROIs. BW and derived BMI were the only two parameters found to be significantly related to bone remodeling in the lateral ROI, since increased long‐term lateral bone resorption was found in heavier and more overweight subjects. Additional simulations of the four tibiae with unknown subject BW using the unscaled load cases (BW of 75 kg) showed a minimal and inconsistent difference in intrasubject 15‐year lateral BMD change compared with considered outcome based on loading following the average female subject BW (59 kg), indicating that effects of BW and BMI are related to associated differences in preoperative bone structure rather than to the extent of the physiological forces. The used implant size and achieved bone coverage were not related to any of the regional BMD measures.

**TABLE 5:** Pearson correlation coefficient between regional baseline and remodeling BMD outcome, and (preoperative) subject parameters

	Pearson correlation coefficient, *ρ*								
	Baseline ROI BMD			15‐year relative ROI BMD change			15‐year net ROI BMD change		
	Medial	Lateral	Distal	Medial	Lateral	Distal	Medial	Lateral	Distal
Baseline BMD									
Medial ROI		0.676^a^	0.758^a^	−0.385	−0.071	0.330	−0.873^a^	−0.161	0.496^a^
Lateral ROI	0.676^a^		0.735^a^	−0.396^a^	−0.095	0.515^a^	−0.662^a^	−0.193	0.640^a^
Distal ROI	0.758^a^	0.735^a^		−0.465^a^	−0.154	0.498^a^	−0.725^a^	−0.267	0.721^a^
Age	0.518^a^	0.276	0.278	−0.173	−0.093	0.426^a^	−0.412^a^	−0.131	0.394^a^
BW	0.357	0.551^a^	0.337	0.127	−0.328	0.377	−0.144	−0.429^a^	0.445^a^
Height	−0.177	0.008	−0.173	−0.192	0.354	0.247	0.026	0.378	0.116
BMI	0.347	0.361	0.356	0.189	−0.450^a^	0.117	−0.119	−0.531^a^	0.244
Implant size	−0.273	0.149	−0.064	−0.255	0.195	0.356	0.101	0.188	0.236
Bone coverage	−0.039	−0.106	−0.048	−0.036	0.160	−0.380	0.024	0.123	−0.309

^a^ Significance for *p* < .05.

## 4 DISCUSSION

In the current study, the results of tibial bone remodeling simulations were compared against a longitudinal clinical DEXA study in a comparable patient group.^25^, ^26^, ^27^ The computational results were also used to investigate the relative effects of patient characteristics and implant alignment. The remodeling simulations predicted medial and lateral bone density changes that were very similar to the clinical results. The outcome also demonstrated the effect of alignment on bone remodeling, particularly in the lateral ROI.

The rate of computational bone remodeling over postoperative time indicated that the vast majority of tibial density changes took place within the first 2–3 years, with the highest remodeling rates found in the initial 6 months after surgery (Figure 4), in line with the bone changes in the clinical population.^25^ Other DEXA studies also reported the greatest bone loss to occur between initial follow‐up time points within 2 years, but found ongoing density changes after 3 years.^3^, ^18^, ^50^ A single‐photon emission CT (SPECT) study suggested implant‐induced remodeling to take place in the first 2 years after uncomplicated TKA, or slightly longer in cases with more extensive bone remodeling, based on leveling of SPECT uptake as a measure of bone metabolic activity.^51^ Ongoing long‐term bone loss is likely caused by systemic bone changes due to aging and physical activity over time, which was not accounted for by the strain‐adaptive remodeling simulations. The extent of computational bone loss over time in the two proximal ROIs was underestimated following the absolute BMD difference with baseline BMD compared with the clinical results (Table 4).

Computational remodeling was performed on a Japanese TKA population different from the Japanese patients in the clinical remodeling study,^25^, ^26^, ^27^ since no preoperative CT scans of the DEXA study were available. No significant differences were encountered in patient demographics of both populations (Table 3). However, preoperative BMD was significantly lower in the medial ROI over the FE tibiae. Several densitometry‐related factors could have contributed to this BMD difference. Firstly, the clinical scans included soft tissue structures around the bone, which contributed to the total projected density, while the projected FE densities were based on only the segmented tibiae, resulting in lower BMD as encountered in the proximal ROIs. And secondly, density differences could have been caused by the use of a different image modality in the two populations; although a very good correlation had been found between HU measured in CT and DEXA‐derived BMD,^52^ and in the scan‐specific linear fit between HU and cubic BMD,^39^ any deviations could be amplified over both established relations. Moreover, considerable speckle noise was encountered throughout CT images of the FE subjects, affecting the histogram‐based air–fat–muscle calibration,^39^ in addition to the direct effect of speckle on voxel intensities; the calibration was performed using only the air and muscle values in clinical scans in which no explicit fat tissue intensity peak was observed due to the speckle, making the HU–BMD relation more prone to deviations.

The significant increase in proximal BMD in the clinical population at 2 weeks after TKA relative to 2 weeks preoperatively was suggested to be predominantly caused by presence of bone cement in the ROIs, since a larger two‐week postoperative increase in regional BMD was reported over the subjects with a cemented Sigma RP tray than in a similar subject group receiving an uncemented trabecular metal component.^25^ Densification due to damage and compaction of bone tissue during TKA probably also contributed to the initial postoperative BMD increase, as a significant lateral increase in the first postoperative measurement relative to preoperative baseline was reported following different designs of cementless tibial components as well.^25^, ^53^ It is highly unlikely that any structural bone remodeling had a considerable contribution to the measured densification, considering the extent of BMD change observed over only a 4‐week time period. To correct for systematic overestimation of periprosthetic BMD in DEXA scans due to cementation, net BMD changes have been considered over postoperative time, in addition to relative density changes, in Figure 4 and Table 4.

Differences in preoperative BMD between both populations (Table 3) and underestimation of the net bone loss in the computational group (Table 4) could be the result as well of previously reported effects of (potential) differences in patient characteristics (Table 3). Since preoperative joint angles of the clinical population were not reported, it could be that native varus deformation was more prevalent over these subjects, leading to relatively more initial densification in the medial condyle,^6^, ^54^, ^55^ generally increased density over the preoperative proximal tibia,^6^ and increased medial bone loss following TKA.^3^, ^6^, ^7^, ^8^ Considering the average age difference between FE and clinical subjects, it could also be that osteoarthritis (OA) was more progressed over the older clinical TKA population; knee OA has also been related to increased constitutional varus angles and higher local proximal baseline BMD, respectively,^46^, ^56^ although age was not found to be directly related to the knee angles measured in the FE population (Table 5). Higher proximal preoperative BMD was related to increased bone loss in a previous clinical study,^6^ which was in line with the differences in net bone loss between the clinical and the FE group (Table 4), and with the correlations between baseline BMD and 15‐year BMD change within the FE group (Table 5).

Despite reported in numerous clinical studies,^3^, ^6^, ^7^, ^8^ no relation was found between measured knee angles and baseline ML density values in the current FE subjects. This could be due to the fact that the alignment angles were measured in supine, non‐weight‐bearing CT scans, which may differ from alignment in a weight‐bearing position.^57^ On the other hand, the difference in sex distribution over the assigned alignment subgroups, with 71% of male knees considered in preoperative varus versus 42% of female knees, was in line with the finding that constitutional varus is more common in men than in women,^19^ suggesting a reasonable subdivision of the knees over the alignment groups was made.

The effect of tibial alignment on postoperative remodeling was also not significant in the current study, in contrast to a previous computational study.^11^ However, in line with our hypothesis, a reduction in lateral bone loss was still observed in the native varus group (Figure 5), and lateral densification was encountered solely in individual varus tibiae. Most previous remodeling studies only predicted bone loss in the condyle with a reduction of load,^3^, ^8^, ^9^, ^10^, ^11^ while only a few studies reported BMD loss as a result of load increase in the contralateral condyle.^4^, ^6^ Discrepancy in the effect of alignment change on remodeling found over different (computational) studies could be caused by used ROI definitions, preoperative BMD (distributions), and several factors affecting physiological implant loading and strain transfer to the proximal tibia, such as implant geometry, implant type (type of bearing and cruciate ligament retention), and fixation (cementation).

Although proximal simulated remodeling was in line with clinical measurements, extensive bone formation in the distal ROI as not been reported clinically (Figure 4). Distal bone apposition over the FE population was initiated by a large relative increase in local SED observed in cancellous bone around the distal tip of the implant keel. Mechanical load transfer through the distal keel, leading to proximal stress shielding, was also indicated to occur clinically, since clinical bone loss was less extensive in the distal ROI compared with the proximal ROIs (Table 4). The difference in bone response to distal strain increase could be caused by several underlying factors. Within the FE models, the implant was assumed to be perfectly fixed to the bone by a smooth cement layer with a consistent thickness, which might have led to the large concentration of strains located underneath the tip of the keel. Postoperative strain increase is expected to occur at more sites on the interface around the keel after an actual TKA, since the implant–bone interface may not be fully and evenly bonded by the cement,^58^ leading to decrease in peak SED values and subsequent bone formation response. In addition, considerable interdigitation of bone cement into trabecular bone was suggested by the presence of bone cement in the clinical ROIs,^25^ which was demonstrated to decrease bone strains with increasing interdigitation depth,^59^ but has not been modeled in current FE simulations. The application of only TF loading on the FE models also leads to greater discrepancy between clinical and FE remodeling pathways more distally in the bone, since bone strains caused by attached soft tissue structures, such as the patella tendon, posterior cruciate ligament and popliteus muscle, are ignored; therefore, relative postoperative SED change and related bone response are expected to be increasingly overestimated in local bone more distant to the baseplate.

Furthermore, the implemented strain‐adaptive remodeling theory and parameter values have been established and validated around periprosthetic femoral bone changes related to hip implants,^23^, ^24^ in which extensive bone formation due to excessive local strain concentration was not reported. As a result, the current remodeling algorithm was mainly derived from observed femoral bone loss as response to decrease in local strains, and behavior of bone formation was currently assumed to be inversely related to bone resorption. However, net bone resorption was found to occur at a much higher rate than bone (re)formation following long‐term changes in mechanical loading,^60^, ^61^ suggesting a higher lazy zone threshold and decreased sensitivity to be used in case of local SED increase, and additional interactions could take place. For instance, post‐yield bone behavior was not accounted for in the bone response and material properties, while an average compressive strain increase of 6.9 and 11.6% in spongy bone of female proximal tibiae were reported for yield and ultimate failure strains, respectively.^62^ To adjust and validate bone behavior in response to strain increase, it would be recommended to use clinical CT scans of longitudinal periprosthetic tibial remodeling studies, enabling reconstruction of preoperative and postoperative subject‐specific FE models and direct comparison of local three‐dimensional clinical and computational bone adaptations over time, to allow for optimization in bone response following underlying strain differences. Such an approach was not possible in the current study, since only longitudinal two‐dimensional DEXA data of the clinical population was available.

## 5 CONCLUSIONS

Based on the comparison between computation bone remodeling outcome and clinical density changes over similar populations, we can conclude that the current strain‐adaptive remodeling algorithm is able to predict the course of bone density changes over time in the proximal tibial ROIs, but not in the distal ROI. Extensive distal bone formation is likely caused by simplifications in implant fixation, (distal) loading conditions and strain‐adaptive theory. To improve computational remodeling, it is recommended to perform further research using intrasubject comparisons, based on longitudinal clinical CT data and physiological load cases including soft tissue representations. Being able to reliably predict tibial periprosthetic remodeling is helpful to guide clinical practice, by identifying risk factors in implant design, surgical technique and tibial features for potential long‐term failure due to excessive regional bone loss.

## Data Availability

Data available on request due to privacy/ethical restrictions.
